# The calcium‐sensing receptor: A novel target for treatment and prophylaxis of neratinib‐induced diarrhea

**DOI:** 10.1002/prp2.521

**Published:** 2019-09-13

**Authors:** Taras Lysyy, Alshad S. Lalani, Elizabeth A. Olek, Irmina Diala, John P. Geibel

**Affiliations:** ^1^ Department of Surgery Yale University School of Medicine New Haven CT USA; ^2^ Puma Biotechnology, Inc. Los Angeles CA USA; ^3^ Department of Cellular and Molecular Physiology Yale University School of Medicine New Haven CT USA

**Keywords:** calcium, electrolytes, fluid secretion, HER2, Nerlynx™

## Abstract

Diarrhea is one of the most commonly reported adverse effect of hemotherapy and targeted cancer therapies, such as tyrosine kinase inhibitors (TKI), which often significantly impact patient quality of life, morbidity, and mortality. Neratinib is an oral, irreversible pan‐HER tyrosine kinase inhibitor, which is clinically active in HER2‐positive breast cancer. Diarrhea is the most common side effect of this potent anticancer drug and the reasons for this adverse effect are still largely unclear. We have recently shown that activation of the calcium‐sensing Receptor (CaSR) can inhibit secretagogue‐induced diarrhea in the colon, therefore we hypothesized that CaSR activation may also mitigate neratinib‐induced diarrhea. Using an established ex vivo model of isolated intestinal segments, we investigated neratinib‐induced fluid secretion and the ability of CaSR activation to abate the secretion. In our study, individual segments of the rat intestine (proximal, middle, distal small intestine, and colon) were procured and perfused intraluminally with various concentrations of neratinib (10, 50, 100 nmol L^−1^). In a second set of comparison experiments, intraluminal calcium concentration was modulated (from 1.0 to 5.0 or 7.0 mmol L^−1^), both pre‐ and during neratinib exposure. In a separate series of experiments R‐568, a known calcimimetic was used CaSR activation and effect was compared to elevated Ca^2+^ concentration (5.0 and 7.0 mmol L^−1^). As a result, CaSR activation with elevated Ca^2+^ concentration (5.0 and 7.0 mmol L^−1^) or R‐568 markedly reduced neratinib‐induced fluid secretion in a dose‐dependent manner. Pre‐exposure to elevated luminal calcium solutions (5.0 and 7.0 mmol L^−1^) also prevented neratinib‐induced fluid secretion. In conclusion, exposure to luminal neratinib resulted in a pronounced elevation in fluid secretion in the rat intestine. Increasing luminal calcium inhibits the neratinib‐associated fluid secretion in a dose‐dependent manner. These results suggest that CaSR activation may be a potent therapeutic target to reduce chemotherapy‐associated diarrhea.

**Abbreviations**
CaSR: calcium‐sensing receptor
EGFRs: epidermal growth factor receptors
FDA: federal drug administration
FITC: fluorescein isothiocyanate
HER: human epidermal growth factor receptor
TKI: tyrosine kinase inhibitors

## 1 INTRODUCTION

Neratinib (PB‐272; also called HKI‐272, Nerlynx™) is an oral anticancer drug, which has clinical activity in HER2‐positive breast cancer^1^ and is the first anticancer drug approved by the US FDA for extended adjuvant treatment of early stage of this type of cancer following trastuzumab therapy. The mechanism of action of neratinib is well studied and described in recent literature.^2^, ^3^ The drug is a potent, small molecule, irreversible tyrosine‐kinase inhibitor of human epidermal growth factor receptors (EGFRs, also known as HER‐1, ErbB‐1), HER2 (neu, ErbB‐2), and HER4 (ErbB‐4).^1^, ^4^, ^5^

Although neratinib has a significant clinical anticancer effect, it also has the commonly reported side effect of diarrhea, 88%‐91% of the patients have reported diarrheal symptoms,^6^, ^7^ particularly 40%—grade 3, and <1%—grade 4.^2^ In grade 4 patients, there is the need for urgent intervention including hospitalization,^8^ significantly reducing quality of life and increasing mortality.

As with all anticancer therapies, an improved treatment strategy for the agent‐associated diarrhea needs to be developed to allow expanded use of these drugs while maintaining the quality of life of the patients. At present loperamide and oral rehydration have been used to improve outcomes and reduce diarrhea.^9^, ^10^ The efficacy of additional antidiarrheal interventions, such as Budesonide and Colestipol, is currently being tested clinically. While these therapies have shown promising effects in preventing neratinib‐induced diarrhea, there is still an unmet outstanding need to have alternative therapies that can further abate this side effect. A better understanding of the biological mechanism(s) that causes diarrhea is also warranted.

The extracellular calcium‐sensing receptor (CaSR) is a member of the G‐protein‐coupled receptor family and has been shown to have a variety of cellular and organ effects across a broad variety of species.^11^, ^12^, ^13^, ^14^, ^15^, ^16^ Our group has previously shown that activation of the CaSR in the colon has been very successfully in abating secretagogue‐induced diarrhea.^14^, ^15^ Furthermore, CaSR has an extremely important physiological role in calcium homeostasis,^17^, ^18^ maintenance of fluid balance,^19^, ^20^ and osmotic regulation.^21^Previous studies have shown that there is high expression of CaSR along the mammalian gastrointestinal tract,^18^, ^22^, ^23^ specifically in the intestinal epithelium,^24^ enteric nerves,^13^ and even on the surface of inflammatory cells.^25^, ^26^ The primary ligand for the receptor is ionized calcium (Ca^2+^_o_) which binds to the surface of the two large exofacial loops of the receptor leading to activation of the receptor leading to decreases in intestinal secretion, motility, inflammation, and increased ionic and fluid absorption.

In the present study, we hypothesized that the activation of CaSR by elevating luminal intestinal calcium could reduce or abate the neratinib‐induced diarrhea. Furthermore, we studied prophylactic pretreatment with oral calcium to prevent neratinib‐induced fluid secretion when the drug was given post prophylactics.

## 2 MATERIAL AND METHODS

### 2.1 Material

#### 2.1.1 Animals

The small intestines were harvested from adult male Sprague‐Dawley rats (250‐365 g body weight, n = 58. All animals were obtained from Charles River Laboratories (Wilmington) and housed in climate‐ and humidity‐controlled 12:12‐hour light‐dark cycled rooms at the Yale animal facility. All animals were fed standard rodent chow and then fasted for 12‐15 hours prior to the study with continued free access to water.

#### 2.1.2 Perfusion solutions

For our study, we used a Ringer HEPES buffer solution (HEPES solution) with different Ca^2+^ concentrations (see Table 1) with a final pH of 7.4 at 37°C.

**Table 1:** Composition of the solutions

Solution	Composition of solution	pH
1 mmol L^−1^ Ca^2+^ HEPES (Standard HEPES)	117 mmol L^−1^ NaCl, 5 mmol L^−1^ KCl, 1 mmol L^−1^ CaCl_2_*2H_2_O, 1.2 mmol L^−1^ MgSO_4_*7H_2_O, 32.2 mmol L^−1^ HEPES, 10 mmol L^−1^ Glucose.	7.4 at 37°C
5 mmol L^−1^ Ca^2+^ HEPES	109 mmol L^−1^ NaCl, 5 mmol L^−1^ KCl, 5 mmol L^−1^ CaCl_2_*2H_2_O, 1.2 mmol L^−1^ MgSO_4_*7H_2_O, 32.2 mmol L^−1^ HEPES, 10 mmol L^−1^ Glucose.	7.4 at 37°C
7 mmol L^−1^ Ca^2+^ HEPES	105 mmol L^−1^ NaCl, 5 mmol L^−1^ KCl, 7 mmol L^−1^ CaCl_2_*2H_2_O, 1.2 mmol L^−1^ MgSO_4_*7H_2_O, 32.2 mmol L^−1^ HEPES, 10 mmol L^−1^ Glucose.	7.4 at 37°C
Cold HEPES	117 mmol L^−1^ NaCl, 5 mmol L^−1^ KCl, 1 mmol L^−1^ CaCl_2_*2H_2_O, 1.2 mmol L^−1^ MgSO_4_*7H_2_O, 32.2 mmol L^−1^ HEPES, 10 mmol L^−1^ Glucose.	7.4 at 5°C

Note

The osmolality of all solutions was adjusted to 300 (±5) mOsm.

#### 2.1.3 Reagents used

Neratinib was provided by Puma Biotechnology. (PB‐272; Puma Biotechnology Inc.) The drug was dissolved in Poly Ethylene Glycol (Sigma) and added to the Ringer solution to obtain a final concentration of 100 nmol L^−1^, or the concentration indicated in the figures. The pH was maintained at 7.4, osmolality at 300 (±5) mOsm and temperature at 37°C during all experiments.

For all studies that contained neratinib a final concentration of 100 nmol L^−1^ was used. This concentration was obtained by diluting a stock solution containing neratinib and Polysorbate 80 (Sigma). R‐568 hydrochloride was purchased from Sigma‐Aldrich. The drug was dissolved in Dimethyl Sulfoxide (DMSO; Sigma‐Aldrich).

#### 2.1.4 FITC‐inulin

To quantitatively evaluate secretory‐absorptive function of the various intestine segments, we have used the isothiocyanate derivative fluorescein isothiocyanate (FITC) coupled to the nonabsorbable volume marker inulin (hereinafter referred to as “FITC‐Inulin”).^27^, ^28^ Inulin is a nonabsorbable sugar with a molecular weight of 3500 Da, which makes it nonpermeable through the intestinal wall. The concentration of FITC‐Inulin (Sigma‐Aldrich, St. Louis, MO) was 50 µmol L^−1^ for all arms of this study.

#### 2.1.5 Intestinal procurement and intestinal perfusion setup

Entire rat small intestine was procured, and three segments were obtained: proximal, middle, and distal each approximately 10 cm in length. The proximal segment (jejunum) was recovered 10‐12 cm distally from the stomach. The distal segment (ileum) was obtained 1‐2 cm proximally from the ileocecal junction. The middle segment was obtained by cutting off 5 cm above and below the midpoint of the residual intestinal segment. The colon segment was obtained proximal to the anal colon junctional interface, in a 10 cm in length. The procured small intestine segments were washed and flushed with normal HEPES Ringer solution at pH 7.4 at 4°C connected to an extracorporeal perfusion device, which has been previously described.^28^, ^29^ Briefly, the system consists of three‐four isolated perfusion chambers, peristaltic pumps, and water bath to maintain temperature in each chamber and for all solutions at 37°C. Each chamber contains two separate perfusion loops: intraluminal and vascular. The intraluminal and vascular compartment have inflow and outflow outlets, which allow continuous intraluminal and vascular perfusion of the intestinal segment by solutions. For intraluminal perfusion, the intestinal segment is attached to the inlet and outlet of the circuit using a suture to maintain a constant flow condition. Depending on the experiment either retrograde or orthograde perfusion was established by orientating the intestinal segment onto the pump connectors. For the basolateral perfusion, the intestine was once attached to the luminal connectors inserted into a water tight outer chamber. The chamber was filled with warm (37°C) buffer solution and then flow was established to the reservoir containing one of the solutions described in Table 1.

#### 2.1.6 Calculation of results

##### Calibration protocol

To calibrate the nanofluorospectrophotometer (Nanodrop 3300, Thermo Fisher Scientific Inc.) for each measurement we performed a series of three standard calibrations curves for each solution that was used as a perfusate. The mean values of these calibration curves were used as the standard curve for that series of studies. Separate calibration curves were conducted for each study and each tissue used.

##### Experimental sample protocol

Each sample luminal perfusate was composed of FITC‐Inulin + the various solutions found in Table 1 with or without neratinib (control). Timed sample collections were made (at 0, 15, 30, 45, 60, 75, and 90 minutes of perfusion, total seven time points per experiment) and samples (10 µL per sample) were then measured with a nanofluorospectrophotometer. Each timed point sample was collected five times and the mean value of the five samples was then recorded. Each study was reported in replicates of five animals per protocol. This yielded an n = 25 measurements/time point/protocol. To calculate the value of each particular time point, samples were collected and then measured and plotted on the calibration curve that was generated on that day. We collected five samples for each time point for each tissue, the mean values for that time point were then calculated and plotted as a single data point with the appropriate error bars. Each replicate animal undergoing the same protocol was also added to the accumulated data and the final values for the tracing presented came from the data collected under these same conditions.

#### 2.1.7 Statistics

All studies presented are calculated for significance and plotted using the GraphPad Prism 7.01 software (GraphPad software Inc.)*.* All bar graphs are plotted as SD, the p values are calculated using a multiple comparisons ANOVA analyses *(*n.s. *P* > 0.05, *P < .05, **P < .01, ***P < .001, ****P < .0001).

#### 2.1.8 Ethical consideration

The animal handling and procurement of small intestine and colon were performed according to approved protocols of the Animal Care and Use Committee at Yale University (Protocol #2015‐10253).

## 3 RESULTS

### 3.1 Modulation in luminal Ca^2+^ concentration in control conditions

**Figure 1:**
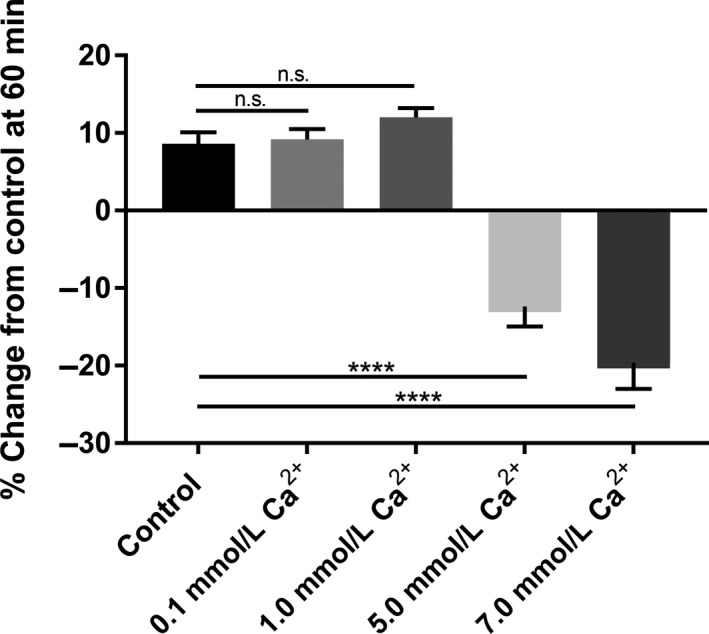
Summary of the basal secretory‐absorptive function of small intestine segments (proximal, middle, and distal) at 60 minutes exposure under various intraluminal calcium concentrations (0.1, 1.0, 5.0, 7.0 mmol L^−1^). Please note that as the concentration is elevated above 1.0 mmol L^−1^ we observe a negative change from control indicative of absorption of fluid and lack of secretion. The data represent the summary of three intestinal segments (proximal, middle, distal) taken from five rats for each calcium concentration at 60 minutes exposure (n = 5)

In this series of studies, we measured the changes in fluid secretion with various concentrations of luminal calcium (See Table 1) compared to normal Ca^2+^ concentrations (1.0 mmol L^−1^ luminal). As shown in Figure 1, we found that increasing the calcium concentration in only the luminal perfusate led to a decrease in the secretion of fluid as indicated by an increase in the FITC‐inulin signal. Further increases in the concentration led to a cessation in secretion and to absorption of fluid as indicated by the increase in fluorescence in the collected perfusate.

### 3.2 Effects of neratinib on the secretory‐absorptive functions of small intestine

**Figure 2:**
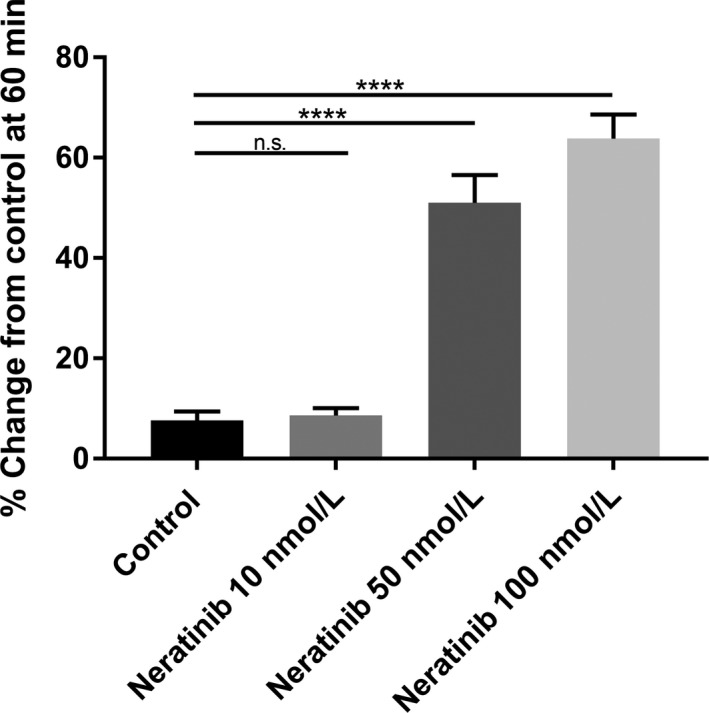
Bar graphs summarizing secretory‐absorptive response of rat small intestinal segments under various intraluminal neratinib concentrations (10, 50, 100 nmol L^−1^). All data are taken at 60 minutes exposure to the drug and represent the summary of three intestinal segments (proximal, middle, and distal) taken from five rats (n = 5). Please note the linear increase secretion with the larger concentrations

After isolation, small intestinal segments were perfused with a solution that contained different concentrations of neratinib (10, 50, 100 nmol L^−1^) and normal Ca^2+^ (1.0 mmol L^−1^ luminal) there was a pronounced and maintained increase in fluid secretion in the small intestine in all segments studied (proximal, middle, and distal) this increased level of fluid secretion was apparent in 15 minutes of exposure to the drug and continued to increase for the entire experimental period (Figure 2).

### 3.3 Effects of elevated luminal Ca^2+^ concentration on neratinib‐induced fluid secretion in small intestine

**Figure 3:**
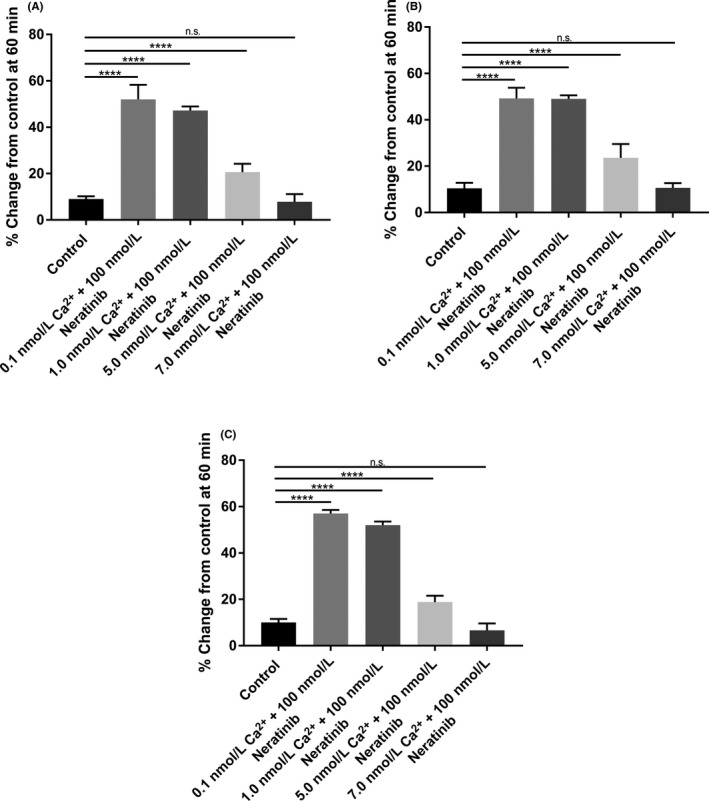
Bar graphs summarizing secretory‐absorptive response of rat small intestinal segments under various intraluminal calcium concentrations (0.1, 1.0, 5.0, 7.0 mmol L^−1^) and in the presence of 100 nmol L^−1^ neratinib. The data are the summary of three segments (proximal, middle, and distal) taken from five rats for each concentration at 60 minutes exposure to the drug (n = 5). (A) Proximal segment of the small intestine; (B) Middle segment of the small intestine; (C) Distal segment of the small intestine

When luminal Ca^2+^ concentration was increased in the presence of 100 nmol L^−1^ luminal neratinib, there was a dose‐dependent cessation in fluid secretion, even in the continued presence of neratinib. As shown in Figure 3A‐C, addition of 5 mmol L^−1^ Ca^2+^ in the lumen is sufficient to inhibit drug‐induced diarrhea in the proximal (Figure 3A), middle (Figure 3B), and distal segments (Figure 3C) of the small intestine. A concentration of 7 mmol L^−1^ Ca^2+^ in the lumen was able to completely block secretion and revert it to normal level in all small intestinal segments (Figure 3). If the concentration was elevated to 7 mmol L^−1^ luminal Ca^2+^ we observed absorption of fluid as shown by elevation in the FITC signal (See Figure 3A‐C). These data show that high luminal Ca^2+^ concentration can also activate the receptor when given concomitantly with the drug and prevent the onset of the secretory surge noted with exposure to the drug.

### 3.4 Effects of neratinib on secretory‐absorptive functions of colon

**Figure 4:**
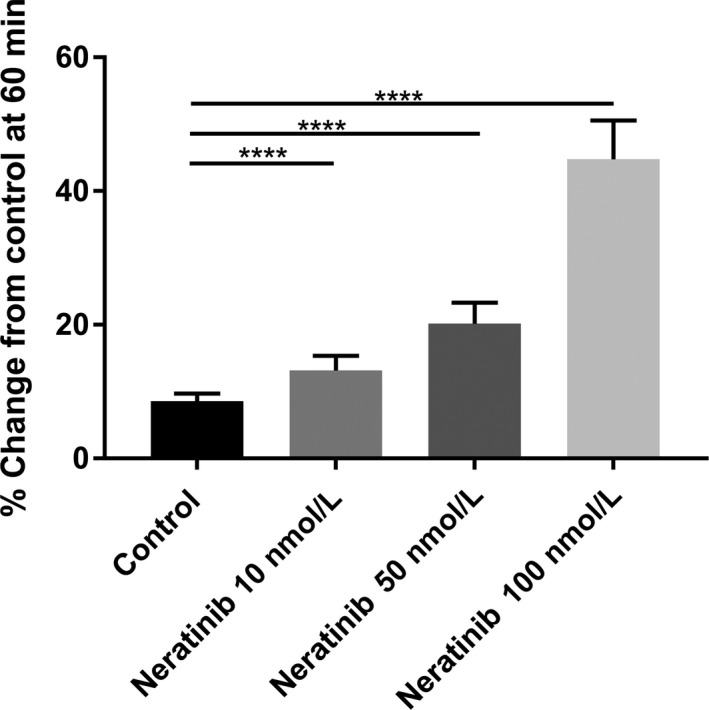
Bar graphs summarizing secretory‐absorptive response of rat colon segment under various intraluminal neratinib concentrations (10, 50, 100 nmol L^−1^). All data are taken at 60 minutes exposure to the drug. The data represent the summary of five colon segments taken from five rats for each drug concentration at 60 minutes exposure (n = 5)

Previous studies from our group have shown that the colon also contains an active CaSR that can be stimulated by elevating the Ca^2+^ concentration in the lumen, or via exposure to a calcimimetic.^13^, ^14^, ^18^, ^24^, ^30^ In this series using isolated colons from rats we were able to see a rapid and sustained fluid secretion following exposure to increasing doses of neratinib (Figure 4).

### 3.5 Elevated luminal Ca^2+^ concentration effects on luminal neratinib exposure in colon

**Figure 5:**
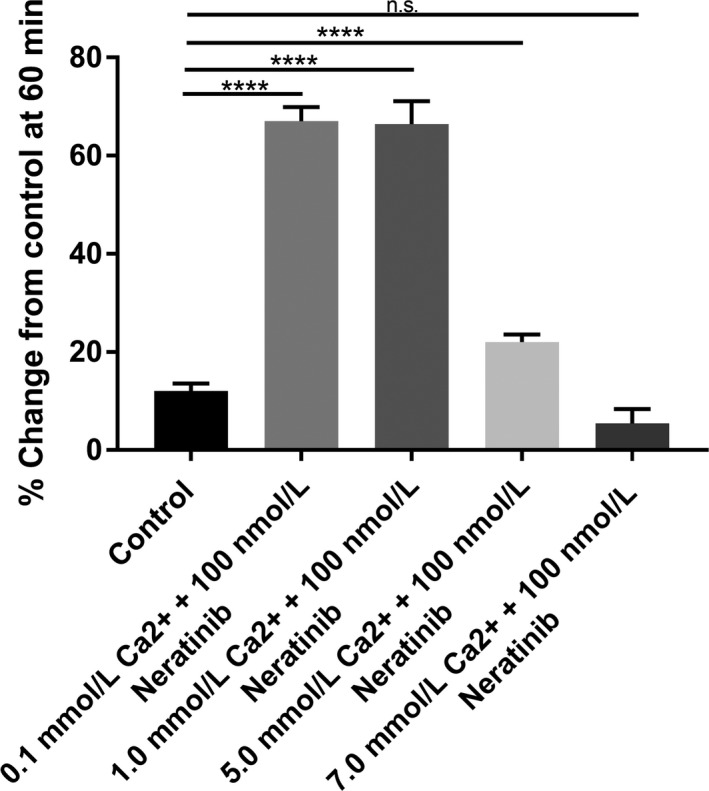
Bar graphs summarizing secretory‐absorptive response of rat colon segment under various intraluminal calcium concentrations (0.1, 1.0, 5.0, 7.0 mmol L^−1^) and in the presence of 100 nmol L^−1^ neratinib. The data are the summary of five colon segment taken from five rats for each calcium concentration at 60 minutes exposure to the drug (n = 5)

These studies were designed to determine if CaSR activation via elevated levels in luminal Ca^2+^ (5 and 7 mmol L^−1^) could block neratinib‐induced fluid secretion in the colon. As shown in Figure 5 the addition of 5 mmol L^−1^ Ca^2+^ to the luminal perfusate reduced the neratinib secretion to basal fluid secretion levels in the colon. Interestingly, when 7 mmol L^−1^ Ca^2+^ was added to the lumen we observed that fluid absorption occurred even in the presence of neratinib.

### 3.6 Memory effects of CaSR activation prior to neratinib exposure

**Figure 6:**
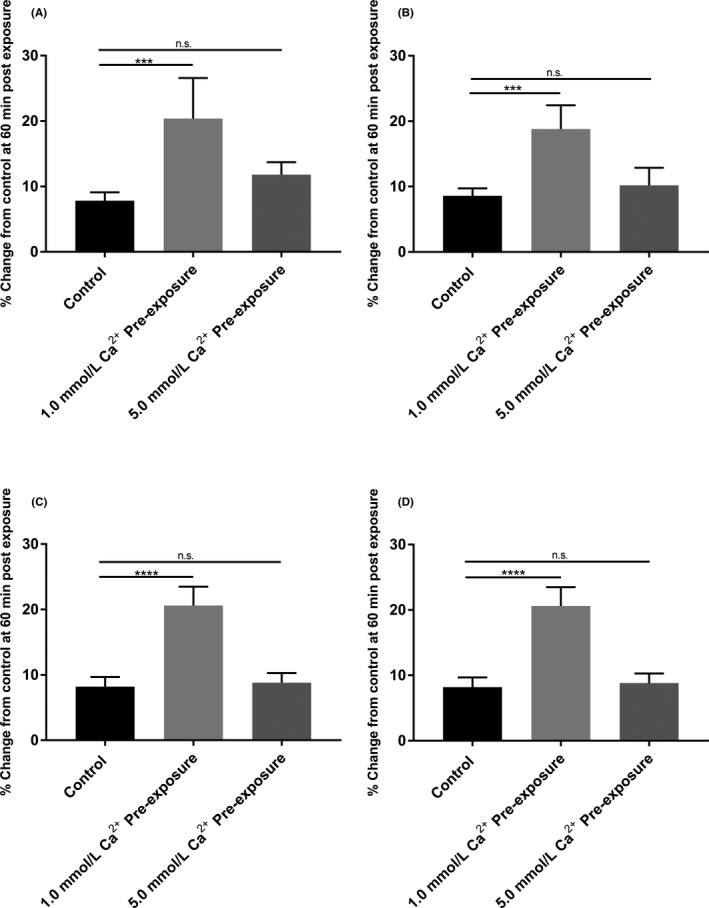
Bar graphs summarizing secretory‐absorptive response of rat small intestinal and colon segments with 15 minutes pre‐exposure with elevated Ca^2+^ concentration (5 and 7 mmol L^−1^) in the presence of 100 nmol L^−1^ neratinib and followed by 1 mmol L^−1^ Ca^2+^ concentration in the presence of 100 nmol L^−1^ neratinib. The data are the summary of five segments (proximal, middle, distal, and colon) taken from five rats at 60 minutes exposure to the drug (n = 5). (A) Proximal segment of the small intestine; (B) Middle segment of the small intestine; (C) Distal segment of the small intestine; (D) Colon

Following the positive effects of elevating luminal Ca^2+^ to abate neratinib‐induced fluid secretion we examined pre‐exposure to elevated Ca^2+^ (5 and 7 mmol L^−1^) prior to neratinib exposure. As shown in Figure 6A‐C, 15 minutes of elevated Ca^2+^ followed by a return to 1.0 mmol L^−1^ Ca^2+^ plus neratinib (100 nmol L^−1^) still prevented neratinib‐induced fluid secretion in the proximal, middle, and distal segments of the small intestine for 45 minutes. In a separate series using the colon under the identical conditions (15 minutes pre‐exposure high Ca^2+^: 5 or 7 mmol L^−1^) followed by neratinib exposure with normal 1 mmol L^−1^ Ca^2+^ prevented neratinib‐sensitive fluid secretion (Figure 6D).

### 3.7 Effects of R‐568 (calcimimetic) on neratinib‐induced fluid secretion in the small intestine and colon

**Figure 7:**
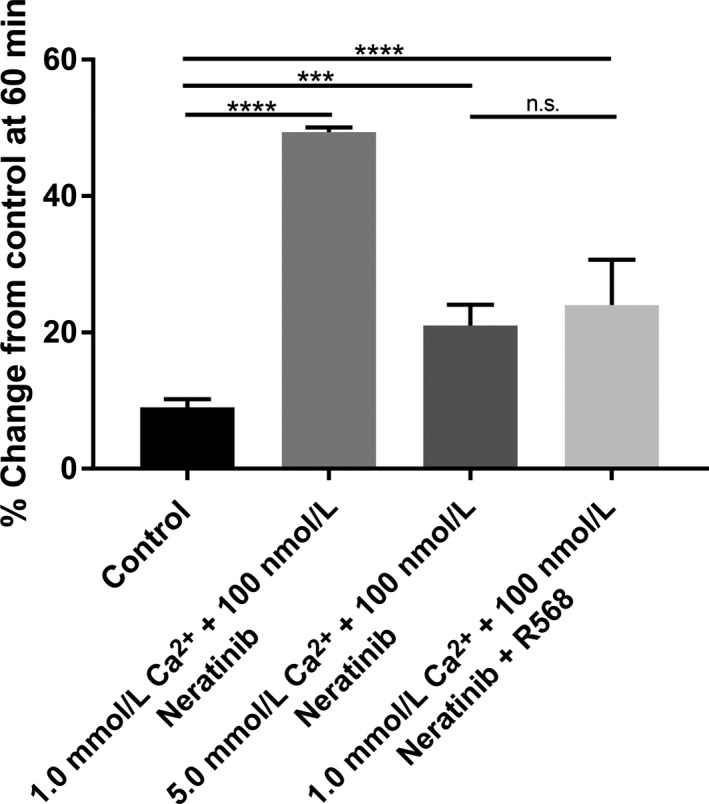
Bar graphs summarizing secretory‐absorptive response of rat small intestine segment under normal luminal Ca^2+^ concentration (1 mmol L^−1^), R‐568 (400 nmol L^−1^) with normal luminal Ca^2+^ concentration (1 mmol L^−1^), and elevated Ca^2+^ concentration (5 mmol L^−1^) in the presence of 100 nmol L^−1^ neratinib. The data are the summary of intestinal segments taken from five rats for each group at 60 minutes exposure to the drug

**Figure 8:**
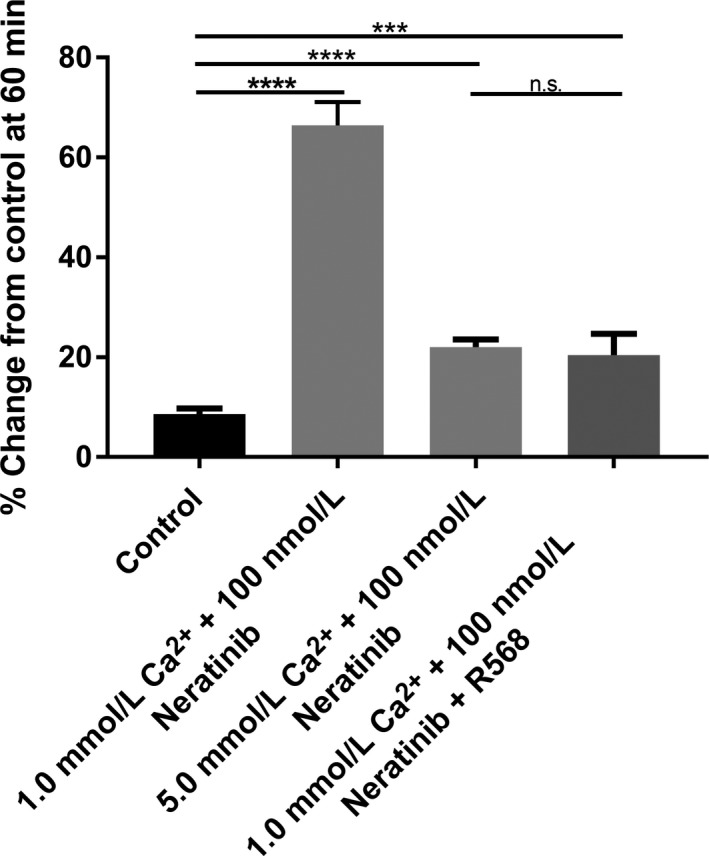
Bar graphs summarizing secretory‐absorptive response of rat colon segments under normal luminal Ca^2+^ concentration (1 mmol L^−1^), R‐568 (400 nmol L^−1^) with normal luminal Ca^2+^ concentration (1 mmol L^−1^), and elevated Ca^2+^ concentration (5 mmol L^−1^) in the presence of 100 nmol L^−1^ neratinib. The data are the summary of colon segments taken from five rats for each group at 60 minutes exposure to the drug

We next compared the effects of R‐568 (400 nmol L^−1^) with a normal luminal Ca^2+^ concentration (1 mmol L^−1^) to elevated Ca^2+^ concentration (5 mmol L^−1^) under 100 nmol L^−1^ Neratinib exposure in both groups. As shown in Figures 7, 8, intraluminal R‐568 with normal Ca^2+^ concentration inhibited Neratinib‐induced secretion at 60 minutes in rat small intestine (See Figure 7) and colon (See Figure 8). The reduction in neratinib‐induced fluid secretion was similar in the calcimimetic with normal luminal Ca^2+^ concentration (1 mmol L^−1^) and elevated Ca^2+^ concentration (5 mmol L^−1^). These data show that R‐568 and high luminal Ca^2+^ concentration can activate the receptor when given concomitantly with the drug and prevent the onset of the secretory surge noted with exposure to the drug.

## 4 DISCUSSION

Targeted therapies such as TKIs are showing promising clinical efficacy in cancer patients, however, the side effects of the therapy can in some cases adverse symptoms that may lead to the cessation of treatment.^31^, ^32^, ^33^, ^34^ A very common adverse effect from chemotherapy or certain TKIs, such as neratinib, is diarrhea which can lead to dramatic loss of fluids and electrolytes, and under severe conditions also lead to malabsorption of nutrients due to the accelerated transit time along the intestine.^35^, ^36^

In the present study, we investigated if activation of the CaSR either concurrently or prophylactically prior to exposure to neratinib could lead to a reduction in the loss of fluid and electrolytes, which would allow patients an improved quality of life while taking the drug.

### 4.1 Isolated intestinal segments and fluid secretion

As the small intestine plays an important role in fluid and electrolyte homeostasis,^27^, ^37^, ^38^ we wished to first investigate if the previously reported side effect of diarrhea was linked to neratinib having a direct action in the small intestine. Recently, our team has developed an isolated perfusion system that allows us to look at proximal, middle, and distal small intestine while sampling the content of the lumen.^27^, ^28^, ^38^ The ability to have isolated intestinal segments in vitro that allow us to measure fluid secretion has been the focus of a variety of studies in our laboratory.^27^, ^38^ During those studies we have found that by using a luminal perfusate that contains FITC‐Inulin we can measure in real time the rate of fluid secretion or absorption in all segments of the intestine.^27^, ^28^ Furthermore, it is also possible to control for leaks or degradation of the tissues by sampling the bath perfusate for detectible FITC‐Inulin fluorescence.

### 4.2 Effects of neratinib on fluid secretion in the small intestine

In these studies, we examined and confirmed that exposure to neratinib in the lumen led to a dose‐dependent increase in fluid secretion in proximal, middle, and distal segments of the small intestine (See Figure 3). This increase in fluid secretion occurred within 15 minutes of exposure to the agent and led to further increases in fluid secretion until a maximal level was reached at 60 minutes of exposure where the level was then maintained (See Figure 6A‐C). These results show an interesting effect of the agent on this segment of the intestine. Since there was an increase in the levels of fluid secretion this could not only result in fluid loss but may also cause a reduction in the absorption of electrolytes.

### 4.3 Effects of neratinib on fluid secretion in the colon

Following our observations in the small intestine we wished to examine if neratinib increased fluid secretion also in the colon, and if this agent had a similar profile that effected fluid and electrolyte homeostasis following introduction into the luminal perfusate in the colon. When neratinib was perfused in the lumen of the colon we saw a similar profile to what had occurred in the small intestine, namely, enhanced fluid secretion that commenced within 15 minutes of exposure to the drug and continued elevation in the level of secretion until a maximal level was reached at 60 minutes (See Figure 4).

### 4.4 Modulation of neratinib‐induced fluid secretion in the colon by CaSR activation

The effects of CaSR activation in the colon are well defined by our laboratory regarding secretagogue‐induced diarrhea.^13^, ^15^, ^23^, ^39^ In this series of experiments, we could show that in the col

on as in the small intestine, elevating luminal Ca^2+^ abated the neratinib‐induced fluid secretion in this segment. This data shows that elevating oral Ca^2+^ will give a protective effect to the entire intestine and colon thereby preventing the associated diarrhea while taking the medication. This promising observation now gives a nutraceutical therapy that should have little to no side effects and could be applied at any point of the chemotherapy cycle.

### 4.5 Memory effects of CaSR activation prior to neratinib exposure

One additional question was whether the beneficial effects of Ca^2+^ could be given prior to the neratinib and still maintain the protective antisecretory effect in the gut. We were in the studies shown in Figure 6 able to demonstrate that exposure for 15 minutes to an elevated Ca^2+^ solution would be sufficient to maintain the antisecretory effects when Ca^2+^ concentration returned to the reduced level and neratinib was added. This observation suggests that a short stimulation of the CaSR can provide a “memory effect” whereby the intestine becomes temporarily resistant to neratinib‐induced diarrhea. This is an important finding in that it suggests that a prophylactic dose of Ca^2+^ followed by neratinib and Ca^2+^ may provide an even more complete prevention.

### 4.6 Effects of R‐568 (calcimimetic) on neratinib‐induced fluid secretion in small intestine and colon

To provide additional evidence of the effect(s) of activation of CaSR on the secretory‐absorptive function rat intestine and colon we used R‐568, a small‐molecule calcimimetic. This data shows that intraluminal exposure to elevated Ca^2+^ concentration or R‐568 with normal Ca^2+^ concentration activate CaSR and have similar effects on reduction of the neratinib‐induced fluid secretion.

In closing, these findings demonstrate that activation of the CaSR by calcium can abate the pharmaceutical induced diarrhea associated with exposure to neratinib and abate the side effects of rapid loss of fluid and electrolytes. Both the prophylactic and acute administration of oral Ca^2+^ with neratinib therapy provides new important opportunities for improved patient outcomes.

## DISCLOSURES

TL and JG have no conflict of interest. AL, EO, and ID are employees of Puma Biotechnology.

## AUTHOR CONTRIBUTIONS

TL was involved in experimental design, conducting the experiments, and writing the manuscript. AL, EO, and ID were involved in data evaluation and editing of the manuscript. JG was involved in all aspects of the experimental design, manuscript writing, and editing.
